# Bis(μ_2_-di­phenyl­phosphinamide-κ^2^ *O*:*O*)bis­[bis(di­phenyl­phosphinamide-κ*O*)lithium] dichloride aceto­nitrile disolvate

**DOI:** 10.1107/S1600536814011076

**Published:** 2014-05-21

**Authors:** Ai-Hong Li, Jun-Ping Han, Jing Li

**Affiliations:** a School of Chemistry and Chemical Engineering, Shanxi University, Taiyuan 030006, People’s Republic of China; b State Key Laboratory of Solid Waste Reuse for Building Materials, No. 69, Jingding North Road, Shijingshan District, Beijing 100041, People’s Republic of China

## Abstract

The asymmetric unit of the title compound, [Li_2_(C_12_H_12_NOP)_6_]Cl_2_·2CH_3_CN, contains one-half of the centrosymmetric dication, one chloride anion and one aceto­nitrile solvent mol­ecule. Each Li atom is coordinated by four O atoms [Li—O 1.891 (3) and 2.025 (3) Å] from the four di­phenyl­phosphinamide ligands in a distorted tetra­hedral geometry. In the crystal, weak N—H⋯Cl hydrogen bonds link the anions and dications into columns extending along [100].

## Related literature   

For reviews of related phospho­rus–nitro­gen transition-metal compounds, see: Roesky & Lucke (1989 ▶); Wong *et al.* (1997 ▶). For the crystal structures of related compounds, see: Oliva *et al.* (1981 ▶); Pisareva *et al.* (2004 ▶).
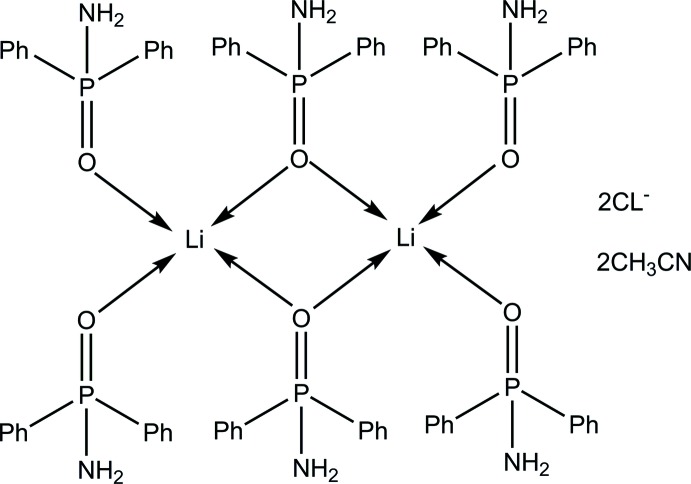


## Experimental   

### 

#### Crystal data  

[Li_2_(C_12_H_12_NOP)_6_]Cl_2_·2C_2_H_3_N
*M* *_r_* = 1470.06
Triclinic, 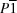

*a* = 11.5625 (7) Å
*b* = 12.5552 (8) Å
*c* = 13.7686 (9) Å
α = 82.559 (1)°
β = 76.515 (1)°
γ = 89.897 (1)°
*V* = 1926.5 (2) Å^3^
*Z* = 1
Mo *K*α radiation
μ = 0.26 mm^−1^
*T* = 296 K
0.30 × 0.25 × 0.20 mm

#### Data collection  

Bruker SMART CCD diffractometer
Absorption correction: multi-scan (*SADABS*; Sheldrick, 1997) ▶ *T*_min_ = 0.925, *T*_max_ = 0.949
13486 measured reflections
6790 independent reflections
5174 reflections with *I* > 2σ(*I*)
*R*_int_ = 0.024

#### Refinement  

*R*[*F* ^2^ > 2σ(*F* ^2^)] = 0.035
*wR*(*F* ^2^) = 0.093
*S* = 1.01
6790 reflections
452 parameters
H-atom parameters constrained
Δρ_max_ = 0.25 e Å^−3^
Δρ_min_ = −0.29 e Å^−3^

### 

Data collection: *SMART* (Bruker, 2000 ▶); cell refinement: *SAINT* (Bruker, 2000 ▶); data reduction: *SAINT*; program(s) used to solve structure: *SHELXS97* (Sheldrick, 2008 ▶); program(s) used to refine structure: *SHELXL97* (Sheldrick, 2008 ▶); molecular graphics: *SHELXTL/PC* (Sheldrick, 2008 ▶); software used to prepare material for publication: *SHELXTL/PC*.

## Supplementary Material

Crystal structure: contains datablock(s) I, New_Global_Publ_Block. DOI: 10.1107/S1600536814011076/cv5450sup1.cif

Structure factors: contains datablock(s) I. DOI: 10.1107/S1600536814011076/cv5450Isup2.hkl

CCDC reference: 1002945

Additional supporting information:  crystallographic information; 3D view; checkCIF report

## Figures and Tables

**Table 1:** Hydrogen-bond geometry (Å, °)

*D*—H⋯*A*	*D*—H	H⋯*A*	*D*⋯*A*	*D*—H⋯*A*
N1—H1*C*⋯Cl1^i^	0.84	2.61	3.4351 (17)	169
N1—H1*D*⋯Cl1^ii^	0.83	2.70	3.4534 (17)	152
N2—H2*C*⋯Cl1^iii^	0.83	2.50	3.2789 (18)	158
N2—H2*D*⋯Cl1^iv^	0.84	2.55	3.3776 (18)	169
N3—H3*C*⋯Cl1^iv^	0.88	2.58	3.3967 (19)	154

Symmetry codes: (i) 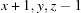; (ii) 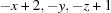; (iii) 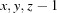; (iv) 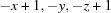.

**Fig. 1.**
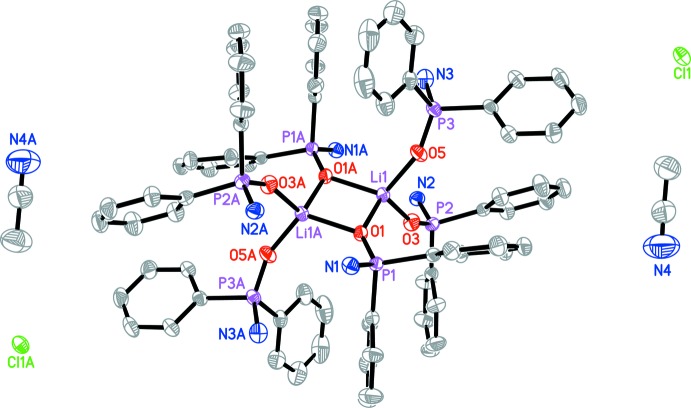
A view of the molecular structure of the title compound, with displacement ellipsoids drawn at the 30% probability level. H atoms and the labels of C atoms were omitted for clarity [symmetry code: (A) 2 - x,-y,-z].

## supplementary crystallographic information

### 1. Comment

The π-electron-rich phosphorus-nitrogen compounds had been known as a type of potential precursors for inorganic polymers with unusual properties, and led to considerable interest in their syntheses and coordination chemistry toward transition metals (Roesky & Lucke, 1989; Wong *et al.*, 1997). The title lithium compound is a by-product in the preparation of this type compounds. Treatment of 1,2-dicyanobenzene with the equivalent LiN(SiMe_3_)_2_ and then the equivalent diphenylphosphinic chloride did not give the π-electron-rich phosphorus-nitrogen compound. The unexpectd title compound was obtained after csystallization in acetonitrile. The crystal structure was ascertained by elemental analysis.

The crystal structure of the compound showed that it has triclinic symmetry. Every lithium ion is coordinated *via* four oxygen of the ligands to give a tetrahedral geometry. The average bond length of Li—O is 1.945 Å. This value is comparable to the analogous lithium compound (Pisareva *et al.*, 2004). The square-plane ring is formed by the two lithium ion and bridged O atoms in which the bond angle of O1—Li1—O1A is 91.28 (17)°. The average bond length of phosphors-nitrogen in the title compound is 1.623 Å. It is very similar to the bond length of phosphors-nitrogen in the crystal structure of diphenylphosphinamide determined in 1981 (Oliva *et al.*, 1981).

### 2. Experimental

All reactions were carried out under nitrogen atmosphere in flamed Schlenk-type glassware on a dualmanifold Schlenk line. n-Butyllithium (1.8 cm^3^, 5 mmol) and NH(SiMe_3_)_2_ (1.06 cm^3^, 5 mmol) were dissolveded in THF (20 cm^3^) at 0°C. The resultant yellow solution was warmed to room temperature and stirred for an additional 2 h. A solution of 1,2-Dicyanobenzene (0.64 g, 5 mmol) in THF (10 cm^3^) was slowly added to the reaction mixture which was stirred at 0°C for two hours before warming up to room temperature. Then diphenylphosphinic chloride (0.95 cm^3^,5 mmol) was added to the mixture at -78°C for an hour before warming up to room temperature and allowed to react overnight. Solvent was then removed in vacuum. The residue was extracted with dichloromethane and the solution was filtered. The solvent of the filtrate was removed in vacuum and was dissolveded in CH_3_CN at room temperature. Finally a coulourless product was obtained. Yield: 0.43 g, 0.83 mmol, 35%. Elemental analysis cacld (%) for C_72_H_72_N_6_O_6_P_6_Li_2_Cl_2_·0.75CH_3_CN·0.25H_2_O: C 65.28, H 5.57, N 6.99; found: C 65.12, H 5.50, N 7.05.

### 3. Refinement

H atoms of phenyl were placed in their idealized positions and allowed to ride on the respective parent atoms with C—H 0.93 Å, and with *U*_iso_(H) = 1.2*U*_eq_. H atoms of acetonitrile were placed in their idealized positions and allowed to ride on the respective parent atoms with C—H 0.96 Å, and with *U*_iso_(H) = 1.5*U*_eq_. H atoms of amino were found from difference Fourier map and N—H bond restraint of 0.84 Å was applied, and with *U*_iso_(H) = 1.2*U*_eq_.

### Crystal data

**Table:**

[Li_2_(C_12_H_12_NOP)_6_]Cl_2_·2C_2_H_3_N	*Z* = 1
*M**_r_* = 1470.06	*F*(000) = 768
Triclinic, *P*1	*D*_x_ = 1.267 Mg m^−^^3^
*a* = 11.5625 (7) Å	Mo *K*α radiation, λ = 0.71073 Å
*b* = 12.5552 (8) Å	Cell parameters from 5610 reflections
*c* = 13.7686 (9) Å	θ = 2.4–26.3°
α = 82.559 (1)°	µ = 0.26 mm^−^^1^
β = 76.515 (1)°	*T* = 296 K
γ = 89.897 (1)°	Block, colourless
*V* = 1926.5 (2) Å^3^	0.30 × 0.25 × 0.20 mm

### Data collection

**Table:**

Bruker SMART CCD diffractometer	6790 independent reflections
Radiation source: fine-focus sealed tube	5174 reflections with *I* > 2σ(*I*)
Graphite monochromator	*R*_int_ = 0.024
ω scan	θ_max_ = 25.0°, θ_min_ = 1.5°
Absorption correction: multi-scan (*SADABS*; Sheldrick, 1997)	*h* = −13→13
*T*_min_ = 0.925, *T*_max_ = 0.949	*k* = −14→14
13486 measured reflections	*l* = −16→16

### Refinement

**Table:**

Refinement on *F*^2^	Primary atom site location: structure-invariant direct methods
Least-squares matrix: full	Secondary atom site location: difference Fourier map
*R*[*F*^2^ > 2σ(*F*^2^)] = 0.035	Hydrogen site location: inferred from neighbouring sites
*wR*(*F*^2^) = 0.093	H-atom parameters constrained
*S* = 1.01	*w* = 1/[σ^2^(*F*_o_^2^) + (0.0401*P*)^2^ + 0.5389*P*] where *P* = (*F*_o_^2^ + 2*F*_c_^2^)/3
6790 reflections	(Δ/σ)_max_ = 0.001
452 parameters	Δρ_max_ = 0.25 e Å^−^^3^
0 restraints	Δρ_min_ = −0.29 e Å^−^^3^

### Special details

**Table:**

Geometry. All e.s.d.'s (except the e.s.d. in the dihedral angle between two l.s. planes) are estimated using the full covariance matrix. The cell e.s.d.'s are taken into account individually in the estimation of e.s.d.'s in distances, angles and torsion angles; correlations between e.s.d.'s in cell parameters are only used when they are defined by crystal symmetry. An approximate (isotropic) treatment of cell e.s.d.'s is used for estimating e.s.d.'s involving l.s. planes.
Refinement. Refinement of *F*^2^ against ALL reflections. The weighted *R*-factor *wR* and goodness of fit *S* are based on *F*^2^, conventional *R*-factors *R* are based on *F*, with *F* set to zero for negative *F*^2^. The threshold expression of *F*^2^ > σ(*F*^2^) is used only for calculating *R*-factors(gt) *etc*. and is not relevant to the choice of reflections for refinement. *R*-factors based on *F*^2^ are statistically about twice as large as those based on *F*, and *R*- factors based on ALL data will be even larger.

### Fractional atomic coordinates and isotropic or equivalent isotropic displacement parameters (Å^2^)

**Table:**

	*x*	*y*	*z*	*U*_iso_*/*U*_eq_	
P1	1.17820 (4)	0.13701 (4)	0.04663 (4)	0.03162 (13)	
O1	1.08254 (11)	0.07098 (10)	0.02456 (9)	0.0363 (3)	
N1	1.30710 (14)	0.08603 (13)	0.04165 (13)	0.0437 (4)	
H1C	1.3528	0.0844	−0.0155	0.052*	
H1D	1.3211	0.0412	0.0874	0.052*	
C1	1.13169 (17)	0.17935 (15)	0.16940 (14)	0.0347 (4)	
C2	1.02395 (18)	0.23041 (17)	0.19186 (16)	0.0444 (5)	
H2A	0.9759	0.2364	0.1460	0.053*	
C3	0.9875 (2)	0.27232 (19)	0.28161 (17)	0.0562 (6)	
H3A	0.9164	0.3085	0.2950	0.067*	
C4	1.0562 (2)	0.2606 (2)	0.35105 (17)	0.0602 (7)	
H4A	1.0315	0.2887	0.4116	0.072*	
C5	1.1608 (3)	0.2079 (2)	0.33147 (17)	0.0609 (7)	
H5A	1.2061	0.1986	0.3794	0.073*	
C6	1.1998 (2)	0.16818 (18)	0.24046 (16)	0.0490 (5)	
H6A	1.2721	0.1339	0.2270	0.059*	
C7	1.20818 (17)	0.25753 (15)	−0.04187 (14)	0.0362 (4)	
C8	1.3130 (2)	0.31763 (18)	−0.05706 (18)	0.0559 (6)	
H8A	1.3701	0.2945	−0.0220	0.067*	
C9	1.3330 (2)	0.4112 (2)	−0.1235 (2)	0.0742 (8)	
H9A	1.4032	0.4514	−0.1328	0.089*	
C10	1.2495 (3)	0.4455 (2)	−0.1764 (2)	0.0720 (8)	
H10A	1.2638	0.5079	−0.2222	0.086*	
C11	1.1448 (3)	0.3874 (2)	−0.16130 (18)	0.0625 (7)	
H11A	1.0879	0.4111	−0.1963	0.075*	
C12	1.1239 (2)	0.29339 (17)	−0.09390 (16)	0.0468 (5)	
H12A	1.0528	0.2544	−0.0837	0.056*	
P2	0.69875 (5)	0.20933 (4)	0.06022 (4)	0.03561 (13)	
O3	0.81395 (12)	0.15618 (11)	0.05724 (11)	0.0479 (4)	
N2	0.59011 (15)	0.15019 (13)	0.03084 (13)	0.0458 (4)	
H2C	0.5927	0.1419	−0.0284	0.055*	
H2D	0.5557	0.0987	0.0726	0.055*	
C13	0.63198 (17)	0.23950 (15)	0.18497 (15)	0.0367 (4)	
C14	0.5256 (2)	0.29275 (18)	0.20447 (17)	0.0512 (6)	
H14A	0.4873	0.3120	0.1527	0.061*	
C15	0.4761 (2)	0.3175 (2)	0.29941 (18)	0.0610 (7)	
H15A	0.4048	0.3533	0.3117	0.073*	
C16	0.5318 (2)	0.2895 (2)	0.37561 (18)	0.0655 (7)	
H16A	0.4990	0.3074	0.4396	0.079*	
C17	0.6362 (2)	0.2350 (2)	0.35852 (18)	0.0700 (8)	
H17A	0.6731	0.2150	0.4111	0.084*	
C18	0.6866 (2)	0.21000 (19)	0.26293 (16)	0.0521 (6)	
H18A	0.7574	0.1733	0.2513	0.063*	
C19	0.72346 (17)	0.33430 (15)	−0.02247 (14)	0.0377 (5)	
C20	0.8251 (2)	0.39536 (17)	−0.02838 (18)	0.0540 (6)	
H20A	0.8786	0.3709	0.0099	0.065*	
C21	0.8479 (2)	0.49210 (19)	−0.0904 (2)	0.0681 (7)	
H21A	0.9160	0.5327	−0.0930	0.082*	
C22	0.7717 (3)	0.52853 (19)	−0.1478 (2)	0.0662 (7)	
H22A	0.7871	0.5940	−0.1893	0.079*	
C23	0.6720 (2)	0.4685 (2)	−0.1442 (2)	0.0715 (8)	
H23A	0.6203	0.4926	−0.1845	0.086*	
C24	0.6473 (2)	0.37226 (19)	−0.08125 (18)	0.0581 (6)	
H24A	0.5784	0.3327	−0.0786	0.070*	
P3	0.81312 (5)	−0.10015 (4)	0.29783 (4)	0.04037 (14)	
O5	0.87404 (14)	−0.02105 (12)	0.21222 (10)	0.0544 (4)	
N3	0.69211 (16)	−0.16261 (16)	0.28927 (14)	0.0554 (5)	
H3C	0.6382	−0.1215	0.2687	0.066*	
H3D	0.7038	−0.2141	0.2565	0.066*	
C25	0.77229 (18)	−0.03918 (16)	0.41151 (14)	0.0402 (5)	
C26	0.8565 (2)	0.02739 (18)	0.43200 (16)	0.0518 (6)	
H26A	0.9291	0.0416	0.3857	0.062*	
C27	0.8337 (2)	0.0727 (2)	0.52018 (18)	0.0631 (7)	
H27A	0.8912	0.1167	0.5335	0.076*	
C28	0.7262 (3)	0.0530 (2)	0.58840 (18)	0.0645 (7)	
H28A	0.7108	0.0839	0.6478	0.077*	
C29	0.6420 (2)	−0.0118 (2)	0.56917 (18)	0.0661 (7)	
H29A	0.5692	−0.0247	0.6156	0.079*	
C30	0.6637 (2)	−0.05871 (19)	0.48111 (16)	0.0535 (6)	
H30A	0.6060	−0.1031	0.4686	0.064*	
C31	0.91044 (19)	−0.20680 (18)	0.32190 (15)	0.0456 (5)	
C32	0.8707 (2)	−0.2984 (2)	0.38919 (18)	0.0624 (7)	
H32A	0.7914	−0.3058	0.4242	0.075*	
C33	0.9491 (4)	−0.3790 (2)	0.4044 (2)	0.0868 (10)	
H33A	0.9222	−0.4405	0.4495	0.104*	
C34	1.0650 (4)	−0.3682 (3)	0.3537 (3)	0.1030 (13)	
H34A	1.1170	−0.4228	0.3640	0.124*	
C35	1.1063 (3)	−0.2777 (3)	0.2874 (3)	0.0959 (11)	
H35A	1.1861	−0.2708	0.2536	0.115*	
C36	1.0290 (2)	−0.1965 (2)	0.27084 (19)	0.0653 (7)	
H36A	1.0567	−0.1353	0.2256	0.078*	
Cl1	0.52785 (5)	0.08079 (5)	0.82677 (4)	0.05126 (16)	
Li1	0.9102 (3)	0.0364 (2)	0.0743 (2)	0.0338 (7)	
C37	0.6237 (4)	0.4221 (4)	0.5809 (3)	0.1075 (12)	
C38	0.5623 (5)	0.3318 (4)	0.6414 (3)	0.1495 (18)	
H38A	0.5694	0.3316	0.7095	0.224*	
H38B	0.5957	0.2677	0.6166	0.224*	
H38C	0.4799	0.3342	0.6396	0.224*	
N4	0.6733 (4)	0.4948 (4)	0.5345 (4)	0.190 (2)	

### Atomic displacement parameters (Å^2^)

**Table:**

	*U*^11^	*U*^22^	*U*^33^	*U*^12^	*U*^13^	*U*^23^
P1	0.0277 (3)	0.0330 (3)	0.0350 (3)	−0.0010 (2)	−0.0076 (2)	−0.0068 (2)
O1	0.0300 (7)	0.0412 (8)	0.0391 (7)	−0.0042 (6)	−0.0064 (6)	−0.0134 (6)
N1	0.0338 (9)	0.0485 (10)	0.0467 (10)	0.0059 (8)	−0.0074 (8)	−0.0030 (8)
C1	0.0375 (11)	0.0321 (10)	0.0342 (10)	−0.0050 (8)	−0.0085 (8)	−0.0035 (8)
C2	0.0412 (12)	0.0518 (13)	0.0423 (12)	−0.0009 (10)	−0.0100 (9)	−0.0144 (10)
C3	0.0518 (14)	0.0628 (15)	0.0524 (14)	−0.0001 (12)	−0.0009 (11)	−0.0228 (12)
C4	0.0794 (19)	0.0614 (16)	0.0369 (13)	−0.0162 (14)	−0.0028 (12)	−0.0157 (11)
C5	0.0848 (19)	0.0636 (16)	0.0404 (13)	−0.0067 (14)	−0.0274 (13)	−0.0057 (12)
C6	0.0555 (14)	0.0513 (13)	0.0444 (13)	0.0034 (11)	−0.0211 (11)	−0.0043 (10)
C7	0.0366 (11)	0.0371 (11)	0.0340 (10)	0.0012 (9)	−0.0050 (9)	−0.0077 (8)
C8	0.0427 (13)	0.0513 (14)	0.0677 (16)	−0.0068 (11)	−0.0073 (11)	0.0040 (12)
C9	0.0607 (17)	0.0562 (16)	0.092 (2)	−0.0126 (13)	−0.0018 (15)	0.0137 (15)
C10	0.093 (2)	0.0471 (15)	0.0616 (17)	0.0027 (15)	0.0004 (15)	0.0115 (12)
C11	0.0855 (19)	0.0569 (16)	0.0498 (14)	0.0174 (14)	−0.0263 (13)	−0.0055 (12)
C12	0.0546 (14)	0.0444 (12)	0.0446 (12)	0.0026 (10)	−0.0181 (10)	−0.0068 (10)
P2	0.0356 (3)	0.0325 (3)	0.0403 (3)	0.0050 (2)	−0.0125 (2)	−0.0040 (2)
O3	0.0442 (8)	0.0468 (8)	0.0541 (9)	0.0159 (7)	−0.0150 (7)	−0.0058 (7)
N2	0.0512 (11)	0.0453 (10)	0.0426 (10)	−0.0075 (8)	−0.0142 (8)	−0.0056 (8)
C13	0.0383 (11)	0.0311 (10)	0.0416 (11)	0.0015 (8)	−0.0119 (9)	−0.0031 (8)
C14	0.0510 (14)	0.0583 (14)	0.0471 (13)	0.0165 (11)	−0.0165 (11)	−0.0080 (11)
C15	0.0592 (15)	0.0651 (16)	0.0564 (15)	0.0216 (13)	−0.0076 (12)	−0.0109 (12)
C16	0.0775 (19)	0.0726 (17)	0.0430 (14)	0.0147 (15)	−0.0046 (13)	−0.0126 (12)
C17	0.0782 (19)	0.093 (2)	0.0424 (14)	0.0209 (16)	−0.0232 (13)	−0.0078 (13)
C18	0.0486 (13)	0.0623 (15)	0.0470 (13)	0.0131 (11)	−0.0152 (11)	−0.0058 (11)
C19	0.0372 (11)	0.0365 (11)	0.0388 (11)	0.0048 (9)	−0.0076 (9)	−0.0059 (9)
C20	0.0544 (14)	0.0439 (13)	0.0661 (16)	−0.0025 (11)	−0.0216 (12)	−0.0026 (11)
C21	0.0652 (17)	0.0464 (15)	0.089 (2)	−0.0149 (13)	−0.0131 (15)	−0.0029 (14)
C22	0.0773 (19)	0.0415 (14)	0.0688 (17)	−0.0003 (13)	−0.0034 (15)	0.0087 (12)
C23	0.0721 (18)	0.0641 (17)	0.0740 (18)	0.0065 (14)	−0.0257 (15)	0.0217 (14)
C24	0.0476 (14)	0.0564 (15)	0.0689 (16)	−0.0031 (11)	−0.0230 (12)	0.0139 (12)
P3	0.0419 (3)	0.0473 (3)	0.0304 (3)	0.0046 (2)	−0.0076 (2)	−0.0010 (2)
O5	0.0662 (10)	0.0590 (10)	0.0312 (8)	0.0009 (8)	−0.0027 (7)	0.0030 (7)
N3	0.0509 (11)	0.0658 (13)	0.0553 (12)	0.0068 (10)	−0.0212 (9)	−0.0136 (10)
C25	0.0447 (12)	0.0420 (12)	0.0309 (10)	0.0076 (9)	−0.0070 (9)	0.0029 (9)
C26	0.0543 (14)	0.0566 (14)	0.0402 (12)	−0.0008 (11)	−0.0027 (10)	−0.0059 (11)
C27	0.0796 (19)	0.0640 (16)	0.0475 (14)	0.0011 (14)	−0.0153 (13)	−0.0136 (12)
C28	0.089 (2)	0.0661 (17)	0.0374 (13)	0.0203 (15)	−0.0096 (13)	−0.0113 (12)
C29	0.0615 (16)	0.0847 (19)	0.0411 (14)	0.0193 (15)	0.0067 (12)	−0.0023 (13)
C30	0.0476 (13)	0.0661 (15)	0.0409 (13)	0.0044 (11)	−0.0018 (10)	−0.0012 (11)
C31	0.0515 (13)	0.0547 (13)	0.0357 (11)	0.0107 (11)	−0.0161 (10)	−0.0135 (10)
C32	0.0811 (18)	0.0611 (16)	0.0476 (14)	0.0170 (14)	−0.0233 (13)	−0.0021 (12)
C33	0.135 (3)	0.073 (2)	0.0621 (18)	0.039 (2)	−0.044 (2)	−0.0075 (15)
C34	0.125 (3)	0.125 (3)	0.079 (2)	0.076 (3)	−0.052 (2)	−0.037 (2)
C35	0.069 (2)	0.146 (3)	0.085 (2)	0.052 (2)	−0.0269 (18)	−0.042 (2)
C36	0.0544 (15)	0.0855 (19)	0.0595 (16)	0.0155 (14)	−0.0153 (13)	−0.0188 (14)
Cl1	0.0512 (3)	0.0653 (4)	0.0356 (3)	0.0019 (3)	−0.0094 (2)	−0.0017 (2)
Li1	0.0315 (17)	0.0380 (18)	0.0317 (17)	0.0030 (13)	−0.0069 (13)	−0.0054 (14)
C37	0.107 (3)	0.103 (3)	0.109 (3)	−0.012 (2)	−0.016 (2)	−0.018 (2)
C38	0.202 (5)	0.141 (4)	0.098 (3)	−0.060 (4)	−0.027 (3)	−0.002 (3)
N4	0.174 (4)	0.132 (3)	0.226 (5)	−0.038 (3)	0.006 (4)	0.012 (3)

### Geometric parameters (Å, º)

**Table:**

P1—O1	1.4928 (13)	C19—C24	1.375 (3)
P1—N1	1.6112 (16)	C19—C20	1.385 (3)
P1—C7	1.795 (2)	C20—C21	1.379 (3)
P1—C1	1.7991 (19)	C20—H20A	0.9300
O1—Li1	1.980 (3)	C21—C22	1.357 (4)
O1—Li1^i^	2.025 (3)	C21—H21A	0.9300
N1—H1C	0.8423	C22—C23	1.366 (4)
N1—H1D	0.8335	C22—H22A	0.9300
C1—C6	1.386 (3)	C23—C24	1.381 (3)
C1—C2	1.388 (3)	C23—H23A	0.9300
C2—C3	1.381 (3)	C24—H24A	0.9300
C2—H2A	0.9300	P3—O5	1.4777 (15)
C3—C4	1.373 (3)	P3—N3	1.6405 (19)
C3—H3A	0.9300	P3—C25	1.795 (2)
C4—C5	1.366 (3)	P3—C31	1.795 (2)
C4—H4A	0.9300	O5—Li1	1.891 (3)
C5—C6	1.386 (3)	N3—H3C	0.8823
C5—H5A	0.9300	N3—H3D	0.8287
C6—H6A	0.9300	C25—C26	1.385 (3)
C7—C12	1.381 (3)	C25—C30	1.390 (3)
C7—C8	1.388 (3)	C26—C27	1.377 (3)
C8—C9	1.377 (3)	C26—H26A	0.9300
C8—H8A	0.9300	C27—C28	1.373 (3)
C9—C10	1.376 (4)	C27—H27A	0.9300
C9—H9A	0.9300	C28—C29	1.363 (4)
C10—C11	1.373 (4)	C28—H28A	0.9300
C10—H10A	0.9300	C29—C30	1.387 (3)
C11—C12	1.387 (3)	C29—H29A	0.9300
C11—H11A	0.9300	C30—H30A	0.9300
C12—H12A	0.9300	C31—C36	1.385 (3)
P2—O3	1.4831 (14)	C31—C32	1.385 (3)
P2—N2	1.6159 (17)	C32—C33	1.385 (4)
P2—C19	1.796 (2)	C32—H32A	0.9300
P2—C13	1.800 (2)	C33—C34	1.357 (5)
O3—Li1	1.892 (3)	C33—H33A	0.9300
N2—H2C	0.8286	C34—C35	1.372 (5)
N2—H2D	0.8393	C34—H34A	0.9300
C13—C18	1.379 (3)	C35—C36	1.386 (4)
C13—C14	1.386 (3)	C35—H35A	0.9300
C14—C15	1.374 (3)	C36—H36A	0.9300
C14—H14A	0.9300	Li1—O1^i^	2.025 (3)
C15—C16	1.363 (3)	Li1—Li1^i^	2.799 (6)
C15—H15A	0.9300	C37—N4	1.119 (5)
C16—C17	1.374 (3)	C37—C38	1.402 (5)
C16—H16A	0.9300	C38—H38A	0.9600
C17—C18	1.385 (3)	C38—H38B	0.9600
C17—H17A	0.9300	C38—H38C	0.9600
C18—H18A	0.9300		
			
O1—P1—N1	118.66 (8)	C21—C20—C19	120.7 (2)
O1—P1—C7	109.85 (8)	C21—C20—H20A	119.6
N1—P1—C7	104.13 (9)	C19—C20—H20A	119.6
O1—P1—C1	110.95 (8)	C22—C21—C20	120.5 (2)
N1—P1—C1	106.11 (9)	C22—C21—H21A	119.8
C7—P1—C1	106.30 (9)	C20—C21—H21A	119.8
P1—O1—Li1	140.12 (12)	C21—C22—C23	119.6 (2)
P1—O1—Li1^i^	131.06 (11)	C21—C22—H22A	120.2
Li1—O1—Li1^i^	88.68 (13)	C23—C22—H22A	120.2
P1—N1—H1C	118.0	C22—C23—C24	120.5 (2)
P1—N1—H1D	122.7	C22—C23—H23A	119.8
H1C—N1—H1D	114.9	C24—C23—H23A	119.8
C6—C1—C2	118.55 (19)	C19—C24—C23	120.7 (2)
C6—C1—P1	123.63 (16)	C19—C24—H24A	119.7
C2—C1—P1	117.76 (15)	C23—C24—H24A	119.7
C3—C2—C1	120.6 (2)	O5—P3—N3	118.56 (9)
C3—C2—H2A	119.7	O5—P3—C25	110.89 (9)
C1—C2—H2A	119.7	N3—P3—C25	105.63 (10)
C4—C3—C2	120.0 (2)	O5—P3—C31	110.65 (10)
C4—C3—H3A	120.0	N3—P3—C31	104.03 (10)
C2—C3—H3A	120.0	C25—P3—C31	106.20 (9)
C5—C4—C3	120.2 (2)	P3—O5—Li1	152.08 (14)
C5—C4—H4A	119.9	P3—N3—H3C	115.8
C3—C4—H4A	119.9	P3—N3—H3D	114.9
C4—C5—C6	120.3 (2)	H3C—N3—H3D	109.1
C4—C5—H5A	119.8	C26—C25—C30	118.8 (2)
C6—C5—H5A	119.8	C26—C25—P3	117.40 (15)
C5—C6—C1	120.3 (2)	C30—C25—P3	123.70 (17)
C5—C6—H6A	119.9	C27—C26—C25	120.7 (2)
C1—C6—H6A	119.9	C27—C26—H26A	119.7
C12—C7—C8	119.0 (2)	C25—C26—H26A	119.7
C12—C7—P1	119.53 (16)	C28—C27—C26	120.0 (2)
C8—C7—P1	121.44 (16)	C28—C27—H27A	120.0
C9—C8—C7	120.5 (2)	C26—C27—H27A	120.0
C9—C8—H8A	119.7	C29—C28—C27	120.1 (2)
C7—C8—H8A	119.7	C29—C28—H28A	120.0
C10—C9—C8	120.1 (2)	C27—C28—H28A	120.0
C10—C9—H9A	120.0	C28—C29—C30	120.6 (2)
C8—C9—H9A	120.0	C28—C29—H29A	119.7
C11—C10—C9	120.0 (2)	C30—C29—H29A	119.7
C11—C10—H10A	120.0	C29—C30—C25	119.7 (2)
C9—C10—H10A	120.0	C29—C30—H30A	120.1
C10—C11—C12	120.1 (2)	C25—C30—H30A	120.1
C10—C11—H11A	120.0	C36—C31—C32	119.4 (2)
C12—C11—H11A	120.0	C36—C31—P3	118.41 (19)
C7—C12—C11	120.3 (2)	C32—C31—P3	122.23 (18)
C7—C12—H12A	119.9	C33—C32—C31	120.1 (3)
C11—C12—H12A	119.9	C33—C32—H32A	120.0
O3—P2—N2	121.04 (9)	C31—C32—H32A	120.0
O3—P2—C19	109.35 (9)	C34—C33—C32	120.1 (3)
N2—P2—C19	104.64 (9)	C34—C33—H33A	120.0
O3—P2—C13	111.06 (9)	C32—C33—H33A	120.0
N2—P2—C13	102.34 (9)	C33—C34—C35	120.8 (3)
C19—P2—C13	107.49 (9)	C33—C34—H34A	119.6
P2—O3—Li1	153.97 (13)	C35—C34—H34A	119.6
P2—N2—H2C	121.0	C34—C35—C36	119.9 (3)
P2—N2—H2D	116.1	C34—C35—H35A	120.1
H2C—N2—H2D	113.2	C36—C35—H35A	120.1
C18—C13—C14	118.9 (2)	C31—C36—C35	119.8 (3)
C18—C13—P2	120.15 (16)	C31—C36—H36A	120.1
C14—C13—P2	120.95 (16)	C35—C36—H36A	120.1
C15—C14—C13	120.8 (2)	O5—Li1—O3	108.65 (16)
C15—C14—H14A	119.6	O5—Li1—O1	110.82 (16)
C13—C14—H14A	119.6	O3—Li1—O1	113.23 (16)
C16—C15—C14	119.9 (2)	O5—Li1—O1^i^	116.04 (16)
C16—C15—H15A	120.1	O3—Li1—O1^i^	115.90 (16)
C14—C15—H15A	120.1	O1—Li1—O1^i^	91.32 (13)
C15—C16—C17	120.4 (2)	O5—Li1—Li1^i^	124.7 (2)
C15—C16—H16A	119.8	O3—Li1—Li1^i^	126.5 (2)
C17—C16—H16A	119.8	O1—Li1—Li1^i^	46.32 (10)
C16—C17—C18	120.0 (2)	O1^i^—Li1—Li1^i^	45.00 (9)
C16—C17—H17A	120.0	N4—C37—C38	178.5 (5)
C18—C17—H17A	120.0	C37—C38—H38A	109.5
C13—C18—C17	120.1 (2)	C37—C38—H38B	109.5
C13—C18—H18A	120.0	H38A—C38—H38B	109.5
C17—C18—H18A	120.0	C37—C38—H38C	109.5
C24—C19—C20	118.0 (2)	H38A—C38—H38C	109.5
C24—C19—P2	123.43 (16)	H38B—C38—H38C	109.5
C20—C19—P2	118.52 (16)		
			
N1—P1—O1—Li1	138.66 (19)	C13—P2—C19—C20	83.06 (18)
C7—P1—O1—Li1	−101.80 (19)	C24—C19—C20—C21	0.9 (3)
C1—P1—O1—Li1	15.4 (2)	P2—C19—C20—C21	−179.74 (19)
N1—P1—O1—Li1^i^	−35.57 (18)	C19—C20—C21—C22	−0.8 (4)
C7—P1—O1—Li1^i^	83.98 (16)	C20—C21—C22—C23	−0.3 (4)
C1—P1—O1—Li1^i^	−158.79 (15)	C21—C22—C23—C24	1.3 (4)
O1—P1—C1—C6	130.81 (17)	C20—C19—C24—C23	0.0 (4)
N1—P1—C1—C6	0.6 (2)	P2—C19—C24—C23	−179.3 (2)
C7—P1—C1—C6	−109.80 (18)	C22—C23—C24—C19	−1.1 (4)
O1—P1—C1—C2	−52.26 (17)	N3—P3—O5—Li1	−23.4 (3)
N1—P1—C1—C2	177.57 (15)	C25—P3—O5—Li1	−145.8 (3)
C7—P1—C1—C2	67.13 (17)	C31—P3—O5—Li1	96.7 (3)
C6—C1—C2—C3	2.2 (3)	O5—P3—C25—C26	−44.58 (19)
P1—C1—C2—C3	−174.93 (17)	N3—P3—C25—C26	−174.24 (16)
C1—C2—C3—C4	−2.1 (3)	C31—P3—C25—C26	75.68 (18)
C2—C3—C4—C5	0.2 (4)	O5—P3—C25—C30	138.18 (18)
C3—C4—C5—C6	1.6 (4)	N3—P3—C25—C30	8.5 (2)
C4—C5—C6—C1	−1.5 (3)	C31—P3—C25—C30	−101.57 (19)
C2—C1—C6—C5	−0.4 (3)	C30—C25—C26—C27	0.6 (3)
P1—C1—C6—C5	176.55 (17)	P3—C25—C26—C27	−176.76 (18)
O1—P1—C7—C12	20.38 (19)	C25—C26—C27—C28	−0.7 (4)
N1—P1—C7—C12	148.45 (16)	C26—C27—C28—C29	0.3 (4)
C1—P1—C7—C12	−99.72 (17)	C27—C28—C29—C30	0.2 (4)
O1—P1—C7—C8	−161.65 (17)	C28—C29—C30—C25	−0.3 (4)
N1—P1—C7—C8	−33.6 (2)	C26—C25—C30—C29	−0.2 (3)
C1—P1—C7—C8	78.25 (19)	P3—C25—C30—C29	177.05 (17)
C12—C7—C8—C9	−0.5 (3)	O5—P3—C31—C36	8.5 (2)
P1—C7—C8—C9	−178.4 (2)	N3—P3—C31—C36	136.90 (18)
C7—C8—C9—C10	−0.6 (4)	C25—P3—C31—C36	−111.89 (19)
C8—C9—C10—C11	1.3 (4)	O5—P3—C31—C32	−171.27 (18)
C9—C10—C11—C12	−0.9 (4)	N3—P3—C31—C32	−42.9 (2)
C8—C7—C12—C11	0.9 (3)	C25—P3—C31—C32	68.3 (2)
P1—C7—C12—C11	178.90 (17)	C36—C31—C32—C33	−0.4 (4)
C10—C11—C12—C7	−0.2 (4)	P3—C31—C32—C33	179.36 (19)
N2—P2—O3—Li1	−36.5 (3)	C31—C32—C33—C34	0.2 (4)
C19—P2—O3—Li1	−158.1 (3)	C32—C33—C34—C35	0.4 (5)
C13—P2—O3—Li1	83.5 (3)	C33—C34—C35—C36	−0.7 (5)
O3—P2—C13—C18	−2.0 (2)	C32—C31—C36—C35	0.1 (4)
N2—P2—C13—C18	128.51 (18)	P3—C31—C36—C35	−179.7 (2)
C19—P2—C13—C18	−121.61 (18)	C34—C35—C36—C31	0.5 (4)
O3—P2—C13—C14	177.75 (17)	P3—O5—Li1—O3	99.5 (3)
N2—P2—C13—C14	−51.71 (19)	P3—O5—Li1—O1	−135.5 (2)
C19—P2—C13—C14	58.17 (19)	P3—O5—Li1—O1^i^	−33.2 (4)
C18—C13—C14—C15	1.1 (3)	P3—O5—Li1—Li1^i^	−85.0 (4)
P2—C13—C14—C15	−178.71 (18)	P2—O3—Li1—O5	−63.1 (4)
C13—C14—C15—C16	0.0 (4)	P2—O3—Li1—O1	173.3 (2)
C14—C15—C16—C17	−1.1 (4)	P2—O3—Li1—O1^i^	69.6 (4)
C15—C16—C17—C18	1.2 (4)	P2—O3—Li1—Li1^i^	121.4 (3)
C14—C13—C18—C17	−1.0 (3)	P1—O1—Li1—O5	−57.1 (3)
P2—C13—C18—C17	178.81 (19)	Li1^i^—O1—Li1—O5	118.6 (2)
C16—C17—C18—C13	−0.1 (4)	P1—O1—Li1—O3	65.3 (2)
O3—P2—C19—C24	141.69 (19)	Li1^i^—O1—Li1—O3	−119.0 (2)
N2—P2—C19—C24	10.6 (2)	P1—O1—Li1—O1^i^	−175.65 (16)
C13—P2—C19—C24	−97.6 (2)	Li1^i^—O1—Li1—O1^i^	0.0
O3—P2—C19—C20	−37.60 (19)	P1—O1—Li1—Li1^i^	−175.65 (16)
N2—P2—C19—C20	−168.64 (17)		

Symmetry code: (i) −*x*+2, −*y*, −*z*.

### Hydrogen-bond geometry (Å, º)

**Table:**

*D*—H···*A*	*D*—H	H···*A*	*D*···*A*	*D*—H···*A*
N1—H1*C*···Cl1^ii^	0.84	2.61	3.4351 (17)	169
N1—H1*D*···Cl1^iii^	0.83	2.70	3.4534 (17)	152
N2—H2*C*···Cl1^iv^	0.83	2.50	3.2789 (18)	158
N2—H2*D*···Cl1^v^	0.84	2.55	3.3776 (18)	169
N3—H3*C*···Cl1^v^	0.88	2.58	3.3967 (19)	154

Symmetry codes: (ii) *x*+1, *y*, *z*−1; (iii) −*x*+2, −*y*, −*z*+1; (iv) *x*, *y*, *z*−1; (v) −*x*+1, −*y*, −*z*+1.
